# The clinical value of xSPECT/CT Bone versus SPECT/CT. A prospective comparison of 200 scans

**DOI:** 10.1186/s41824-017-0024-9

**Published:** 2018-03-19

**Authors:** Iain Duncan, Nicholas Ingold

**Affiliations:** Garran Medical Imaging, 1/2 Garran Place, Garran, ACT 2605 Canberra, Australia

**Keywords:** Bone scintigraphy, Bone scan, Bone SPECT/CT, Bone xSPECT/CT, Musculoskeletal diagnosis

## Abstract

**Purpose:**

To prospectively evaluate the clinical utility of xSPECT/CT Bone, a new reconstruction algorithm for single photon emission tomography (SPECT), and compare it with standard SPECT/CT reconstruction.

**Methods:**

Sequential reporting of SPECT/CT followed by xSPECT/CT images in 200 sequential cases commencing August 2015. Differences between the initial SPECT/CT and the final report (after xSPECT/CT reconstruction) were documented and analysed. 12–18 months after the initial study follow-up, clinical data was sought from a subset of cases in which xSPECT/CT changed the primary diagnosis and imaging correlation undertaken in all patients who subsequently had MRI or CT scans of the same region.

**Results:**

A majority of the 200 cases were related to assessment of musculoskeletal complaints. The final (scan) diagnosis was changed after reviewing the xSPECT/CT images in 40 (20%) of cases. The reporting physician (Iain Duncan) assessed that the xSPECT/CT had provided more diagnostic information in 71% of cases. A total of 470 additional lesions were found, equivalent to 2.4 lesions per case. In 33 cases of imaging follow-up there was a high degree of correlation with bone scan findings and xSPECT correlated better than SPECT in regard to detailed findings. In only 15/40 cases of diagnostic change could the outcome be verified and in 12/15 the xSPECT/CT revised diagnosis was confirmed.

**Conclusions:**

In this observational evaluation xSPECT/CT Bone reconstruction offers identifiable imaging improvements over standard SPECT/CT reconstruction algorithms. xSPECT/CT Bone provides an improvement in diagnostic confidence and identifies a greater number of lesions.

## Background

xSPECT/CT Bone is a recent reconstruction algorithm for single photon emission tomography (SPECT) developed by Siemens Healthineers (USA) which integrates information from the CT scan with the raw data from the bone SPECT/CT acquisition prior to reconstruction, which produces a higher resolution SPECT/CT image, known as xSPECT/CT. This technology is now widely available with recent SPECT/CT systems however, the clinical value of this technology has not been well studied beyond case data and Siemens internal publications. This pilot study was undertaken to help assess its clinical utility in our practice. We had previously participated in the evaluation of SPECT/CT versus planar imaging in a previous (unpublished) evaluation (presented at the 2010 ANZSNM meeting in Auckland, New Zealand). The methodology used for that study had proven simple and the data obtained broadly correlated with other publications showing the additional diagnostic benefits of SPECT/CT over planar and SPECT/CT only imaging (Delbeke et al. 2009; Romer et al. 2006a, 2006b; Schillaci et al. 2004; Utsunomiya et al. 2006a, 2006b; Helyar et al. 2010). The same methodology was chosen for this study in order to evaluate the differences between xSPECT/CT Bone and SPECT/CT over 200 sequential cases commencing August 2015.

## Methods

Commencing in August 2015, 200 sequential patients referred for bone scans who had SPECT/CT imaging undertaken were enrolled into the study. Consent was obtained and bone scans were carried out using a Siemens Intevo SPECT/CT hybrid camera. Both conventional Flash 3D and xSPECT/CT reconstruction were applied after a single SPECT/CT acquisition, targeted to answer the clinical problem or assess abnormalities identified on planar imaging. In the case of metastatic disease, SPECT/CT acquisition was based on whole body scan findings. The dose of tracer was between 750 and 850 MBq for adults and images acquired at 2.5–4 h.

The SPECT/CT acquisition was performed using Siemens Intevo hybrid camera and the following parameters. A 15% energy window at 140 keV with a lower scatter window of 15% and a 256 × 256 matrix. The axial field of view of the camera was 38.7 cm. Thirty 18-s projections acquired over 360 degrees using a non-circular orbit continuous acquisition mode. The gamma camera collimator was a low energy high resolution parallel-hole. Immediately following the SPECT acquisition a CT was acquired for the same field of view as the SPECT with a 512 × 512 matrix, pitch 1.5, 0.8-s rotation time and 2 × 1.5 mm collimation. CARE Dose 4D (Siemens Healthcare) including AEC + DOM was used to keep dose low. Tube current used was 40mAs and tube voltage 110kVp. The total duration for the SPECT/CT acquisition was approximately 12-min.

Two CT image reconstructions were performed. The first using a medium smooth filter (B31s kernel) with a 2 mm slice and 1 mm reconstruction increment used for both the CT attenuation correction and integration with the xSPECT Bone reconstruction. The second using a high-resolution medium sharp filter (B50s) with a 2 mm slice and 2 mm reconstruction increment used for image fusion and display purposes.

Two SPECT reconstructions were performed. The first iteratively utilising CT attenuation correction and Flash3D (Siemens Healthcare) with 8 iterations and 4 subsets. Images were smoothed using a Gaussian filter (8.4 mm at full width half maximum). The second using attenuation correction and xSPECT Bone Enhanced (Siemens Healthcare) with 24 iterations and 2 subsets. Images were smoothed using an integrated setting within the xSPECT reconstruction (10 mm).

**Fig. 1:**
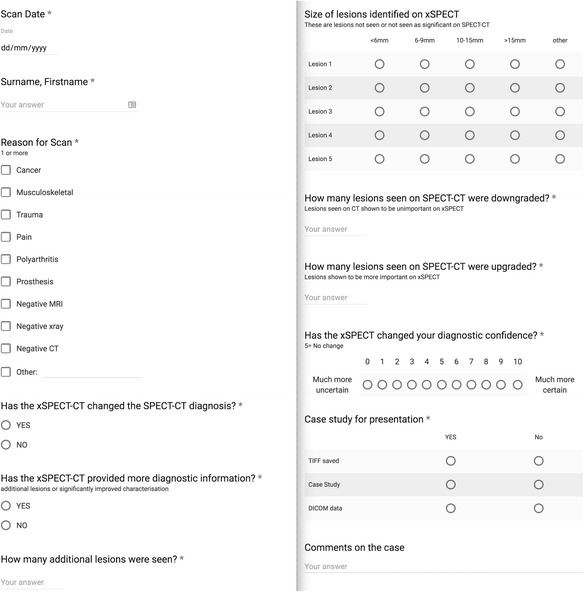
Online form used during the study

Images were then reviewed as 2 mm slices on an intelerad PACS system including a multiplanar fusion module. One of us (Iain Duncan, 20 years of experience) reviewed and reported (with full dictation) the planar and SPECT/CT images, followed later by a second review and revised report using the planar and xSPECT/CT images. All modifications to the initial SPECT/CT report and differences to the (final) xSPECT/CT reports were documented. After the second image review and report process, all changes were documented using an online form (Fig. 1) and synchronised spreadsheet. There were no specific exclusion criteria however patients were not included when reports were issued via teleradiology review only (off-site).

This study was performed in the course of normal solo clinical practice and where any critical findings were made only using the xSPECT/CT reconstruction this information was directly communicated with the referring practitioners.

As this was uncontrolled and unblinded study, verification of the reporting accuracy and diagnostic changes was done retrospectively between July and October 2017. This was done both by seeking clinical follow-up for all patients in which the diagnosis was changed by xSPECT/CT, and by a third-party radiologist reviewing all cases that had either a computerised tomography scan (CT) or Magnetic Resonance Imaging (MRI) study of the same body region within 6 weeks of the bone scan. To assess reliability of the single reviewer and to assess whether the reporting order (SPECT followed by xSPECT) or learning bias was a significant factor, 40 further blinded reads were undertaken more than 18 months after the initial reports. These were randomly selected cases (32 with no diagnosis change and 8 with diagnosis change) selected from a subgroup of 172 of the original 200. 28 of the higher profile cases were excluded to try and avoid recollection bias. Clinical data was restricted to site of pain (if any), no identifying data was included, and planar scans were available for review in all cases. 20 were read SPECT/CT followed by xSPECT/CT, and 20 in the reverse order.

## Results

There were 200 cases (113 females). Based on referral notes and patient information the primary reason for referral were classified as follows: musculoskeletal and or pain 64%, oncology 13%, polyarthritis 10%, prosthesis 7% and trauma 7%. More than 50% of referrals listed more than one reason for referral.

**Fig. 2:**
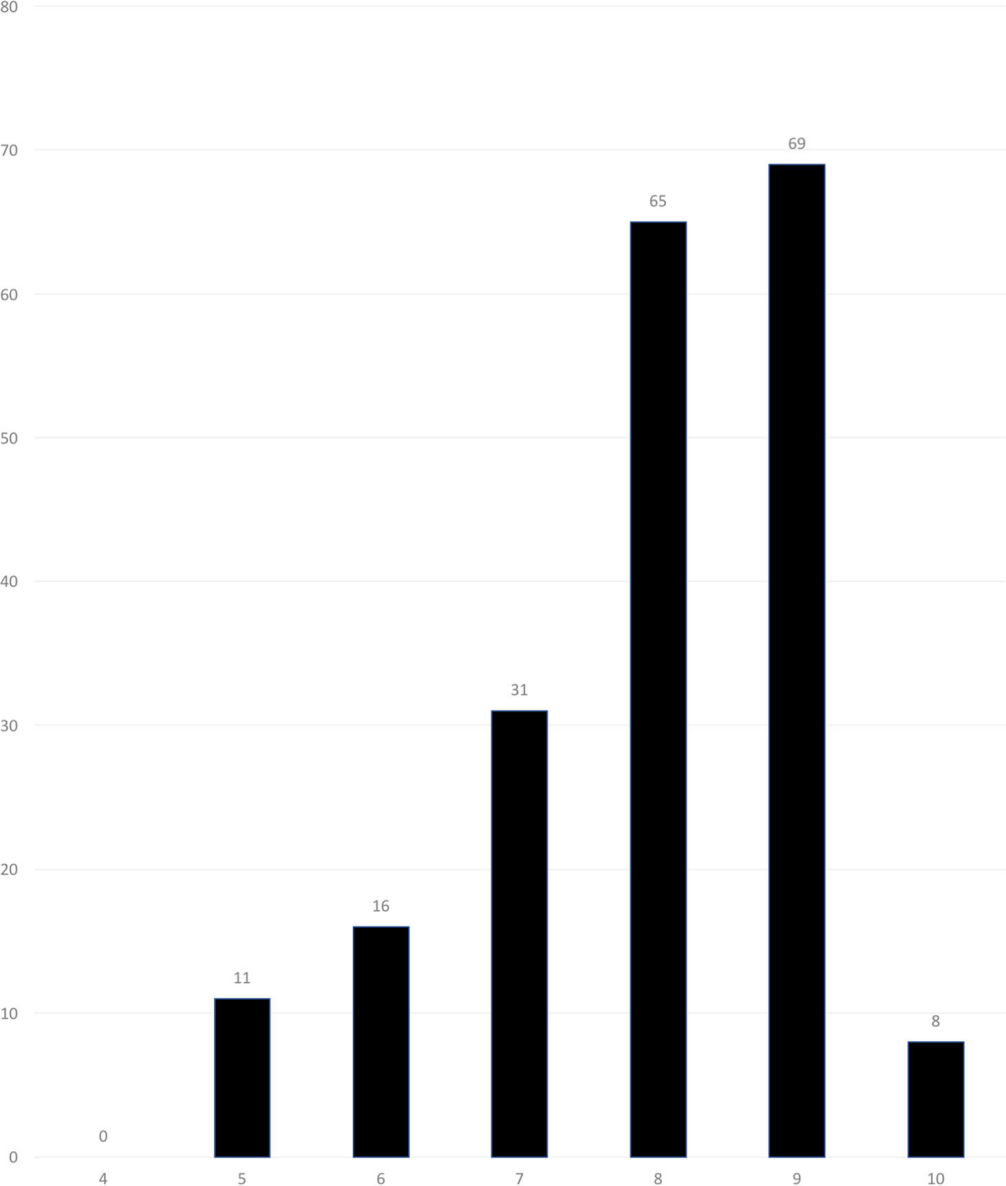
Change in diagnostic confidence with xSPECT. Detailed legend: X-axis: scores of diagnostic confidence 0–10. Score 0–4 indicate a reduced diagnostic confidence, 5 = no change, and 6–10 indicate increasing confidence. Y-axis: Number of patients in each category

The final (scan) diagnosis was changed after reviewing the xSPECT/CT images in 40 (20%) of cases. The reporting physician assessed that the xSPECT/CT had provided more diagnostic information in 71.1% of cases. In 60% of the cases one or more lesions were upgraded by the xSPECT/CT and in 24% of the cases one or more lesions were downgraded. Overall per case there were 1.3 additional significant lesions seen on the xSPECT/CT versus the SPECT/CT. There was 0.3 lesion per case downgraded by the xSPECT/CT. A total of 470 additional lesions were found, equivalent to 2.4 lesions per case. 39% of these lesions measured less than 6 mm, but surprisingly 28% were greater than 15 mm. The reporting physician’s change in diagnostic confidence (score 0–10) after reviewing the xSPECT/CT can be seen in Fig. 2. A score of 8 or more was given in 71% of cases.

**Fig. 3:**
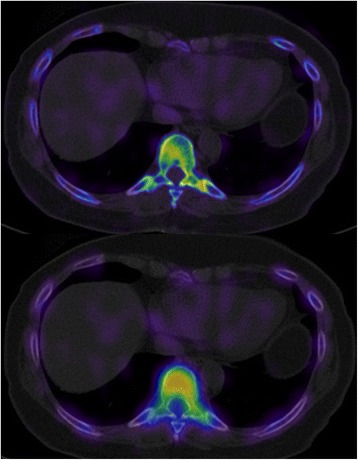
Costovertebral joint uptake in xSPECT/CT v SPECT/CT. Detailed legend: The upper image shows a fused xSPECT-CT transaxial image while the lower image shows a fused SPECT-CT image (same slice in the same patient). Note the clear abnormality identified in the costovertebral joint on xSPECT, which is not identified on SPECT

Of the 40 cases in which xSPECT/CT Bone had changed the diagnosis we were able to contact 21 for clinical follow-up (Table 1). The average follow-up time was 17 months and of those 21 it was only possible to clearly assign outcome in 14. In 6 cases, further information and follow-up had not determined the accuracy of the diagnosis and in one case the diagnosis was confirmed by clinical response and negated by MRI. In the 14 cases where outcome could be assigned the diagnostic change had been confirmed in 12 of 14, either by response to image guided injection (6 cases), additional imaging (3 cases), or indirectly by targeted therapies (3 cases). In the remaining two cases the diagnostic change was incorrect based on a negative response to injection in one case and a normal MRI in the other. In the 40 cases of diagnostic change, 10 were noted to be in the thoracic spine (example Fig. 3) and 2 in the ribs. All involved uptake in locations not evident on SPECT/CT. For larger joints these resolution/uptake changes both enabled and negated diagnoses made on SPECT/CT. In particular the presence or absence of sacroiliitis was both upgraded and downgraded resulting in a change of diagnosis in 8 cases.

**Table 1:** Follow-up data in 21 cases where the xSPECT-CT changed the final report diagnosis

Outcome	Number Cases
Diagnosis confirmed by response to image guided injection	6
Unable to determine accuracy of diagnosis (mixed information)	6
MRI findings support diagnosis	3
Clinical response confirms diagnosis	3
MRI negates diagnosis and agrees with SPECT/CT diagnosis	1
MRI negates but clinical response confirms diagnosis of sacroiliitis	1
xSPECT/CT diagnosis negated by failure of response to image guided injection	1

Thirty-three (16.5%) of the cases had an MRI of the same region within 6 weeks of the bone scan (Table 2). 7 of these were also in the clinical follow-up group with changed diagnoses. 2 cases also had a CT of the same region (both metastatic bone disease) and in both cases the diagnosis was unchanged but the xSPECT/CT showed more lesions than either the SPECT/CT or CT scan. Table 2 shows that there was a high overall correlation between bone scans and MRI (85%) and that this was slightly better for xSPECT/CT in terms of diagnosis and substantially better in regard to anatomic detail in 12 (36%) of the cases. It was noted that in the 33 cases with MRI follow-up there were 2 cases of abnormal sacroiliac uptake seen only on xSPECT/CT but not on SPECT/CT or MRI. It is likely one of these was a false positive but the other was treated successfully with a biologic agent with a diagnosis of axial spondyloarthritis.

**Table 2:** Correlation between bone scan and MRI findings

Outcome	Number Cases
MRI and xSPECT-CT bone scan findings consistent	28 (85%)
MRI and SPECT-CT bone scan findings consistent	26 (79%)
xSPECT/CT significantly better correlation with MRI than SPECT/CT	12 (36%)
xSPECT/CT and SPECT/CT inconsistent with MRI	5 (15%)
xSPECT/CT consistent but SPECT/CT inconsistent with MRI	2 (6%)
Sacroiliitis on xSPECT/CT but negative on SPECT/CT and MRI	2 (6%)
CT, MRI, xSPECT/CT and SPECT/CT findings consistent	2 (6%)

In the reader verification study of 40 cases we found that in none was there a change from the original diagnosis for either SPECT/CT or xSPECT/CT. The number of lesions identified in all SPECT/CT studies was unchanged (regardless of read order). In the xSPECT/CT studies there were 12 additional lesions (0.3 per case) identified and 4 lesions not identified (0.1 per case). None of these lesions was of diagnostic significance and occurred equally in cases where the xSPECT/CT was read first or second. In one case the reader recalled the original and another case was substituted.

**Fig. 4:**
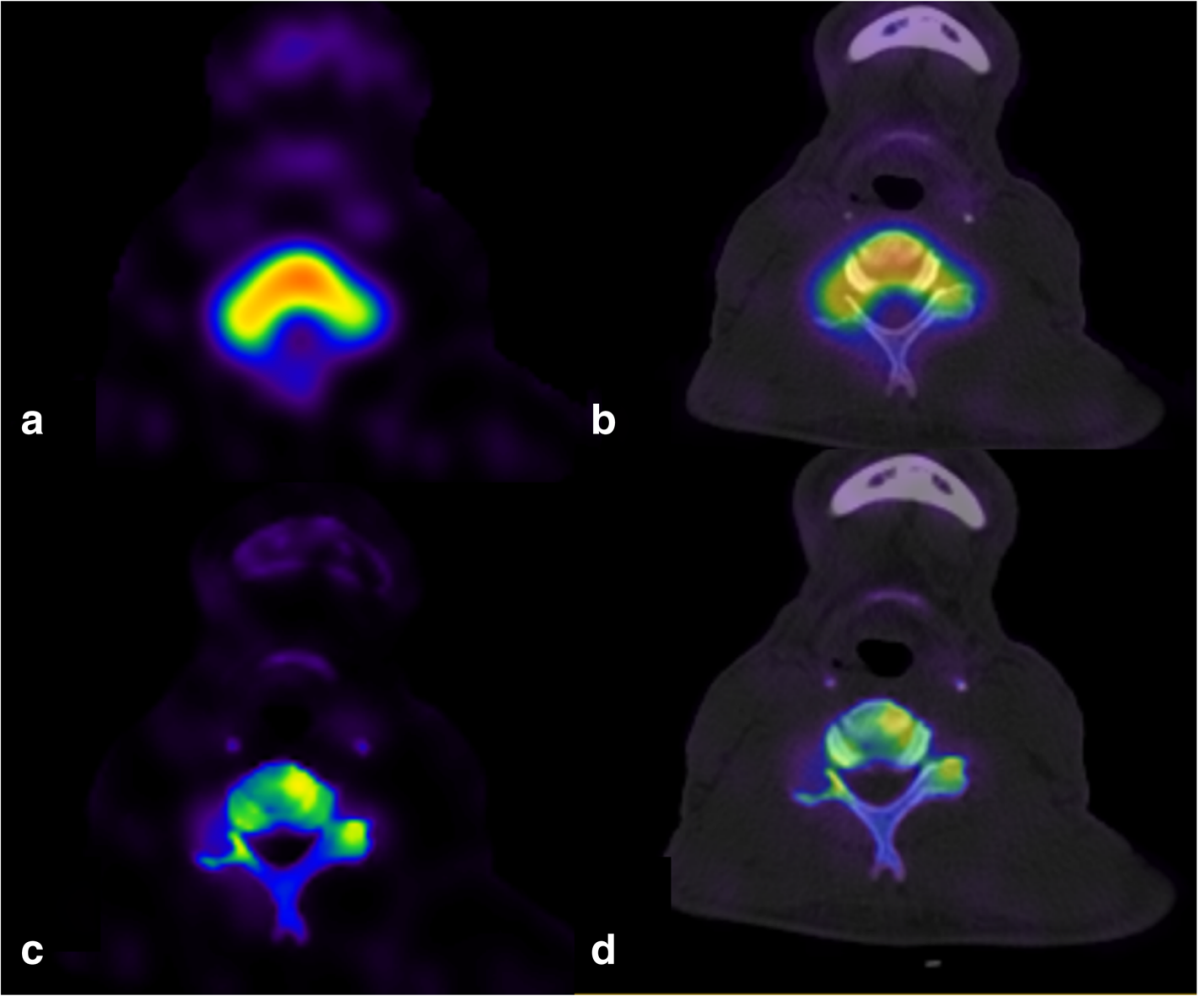
Patient presented with neck pain for evaluation. Detailed Legend: Subsequent xSPECT and SPECT fusion at the C6 level are shown. **a** SPECT, **b** SPECT/CT, **c** xSPECT, and **d** xSPECT/CT. Final diagnosis was metastatic adenocarcinoma of the lung

**Fig. 5:**
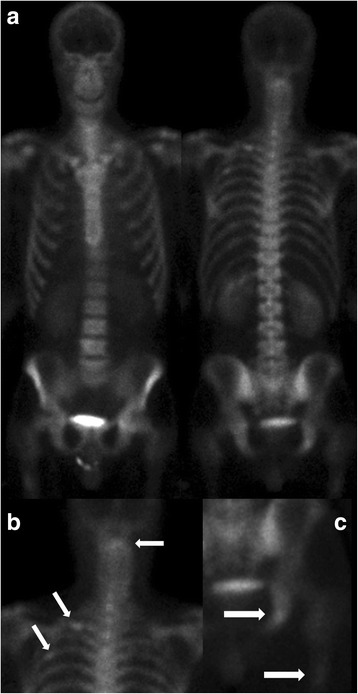
Same patient as in Fig. 4. Detailed Legend: (**a**) Whole body sweep images and (**b**), and (**c**) enlargements, show with arrows showing slightly abnormal uptake in left posterior second and fourth ribs, at the upper cervical spine, in the proximal right femur, and in the right ischium

Specific cases demonstrated the increase in sensitivity of xSPECT/CT Bone. Figure 3 demonstrates the clear benefit of xSPECT/CT in visualising uptake in small joints, most particularly in the thoracic spine. Figure 4 shows how xSPECT/CT outlines cold areas much better than SPECT, in this case a metastatic lesion in the C6 pedicle in a patient who presented with neck pain. In this patient, the whole-body sweep images (Fig. 5) show several subtle abnormalities, the SPECT/CT several more, and the xSPECT/CT several more again.

**Fig. 6:**
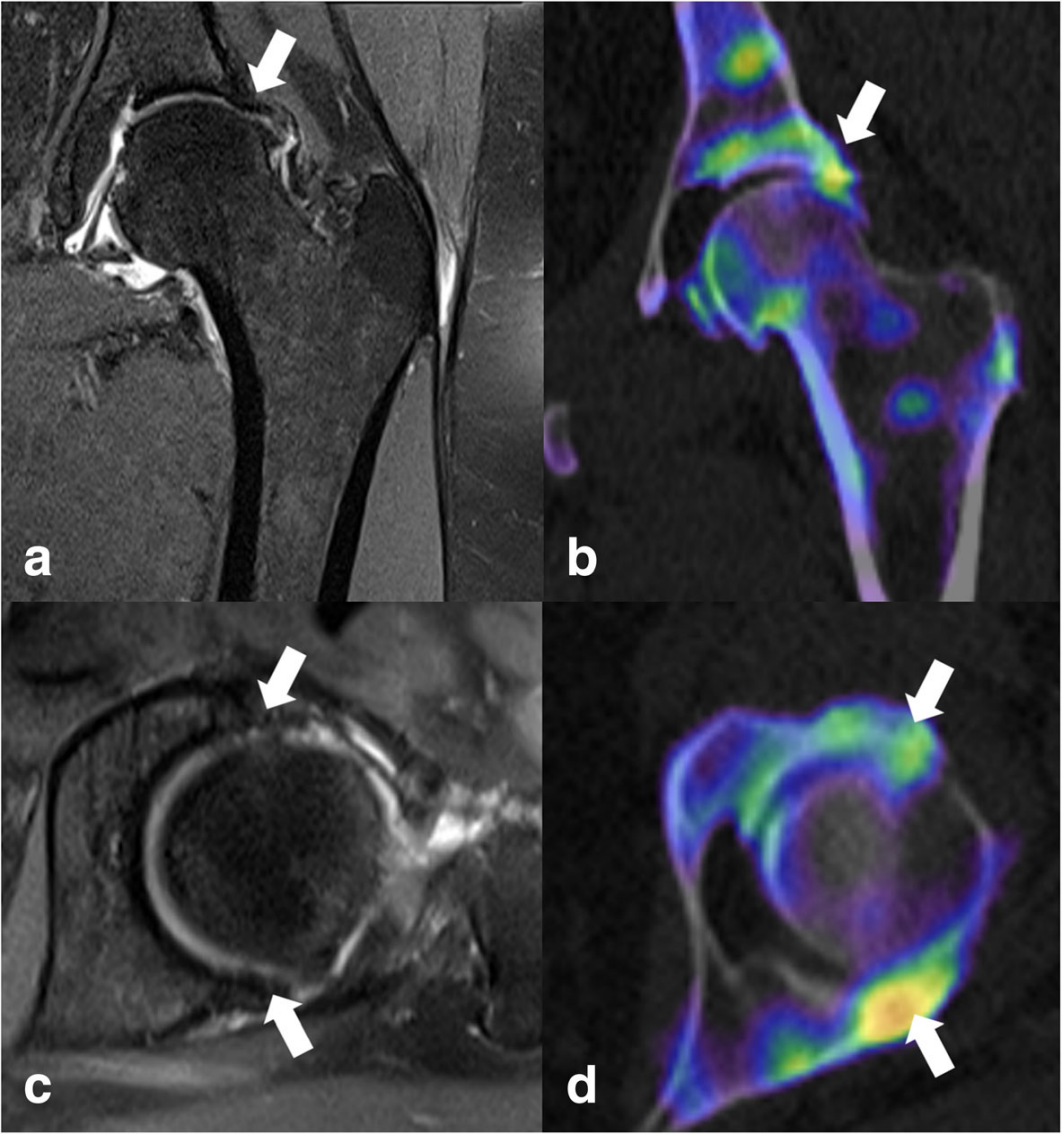
MRI and xSPECT/CT of an osteoarthritic left hip. Detailed Legend: Corresponding slices from the same patient: **a** Coronal T2 fat suppressed image, **b** Coronal xSPECT-CT image, **c** Axial T2 fat suppressed image and **d** Axial xSPECT-CT images in the same patient. Thick arrows show cartilage loss (MRI) with corresponding adjacent bone changes (xSPECT/CT)

An example of the better MRI correlation with xSPECT/CT Bone than SPECT/CT is shown in a case of hip osteoarthritis (Fig. 6).

**Fig. 7:**
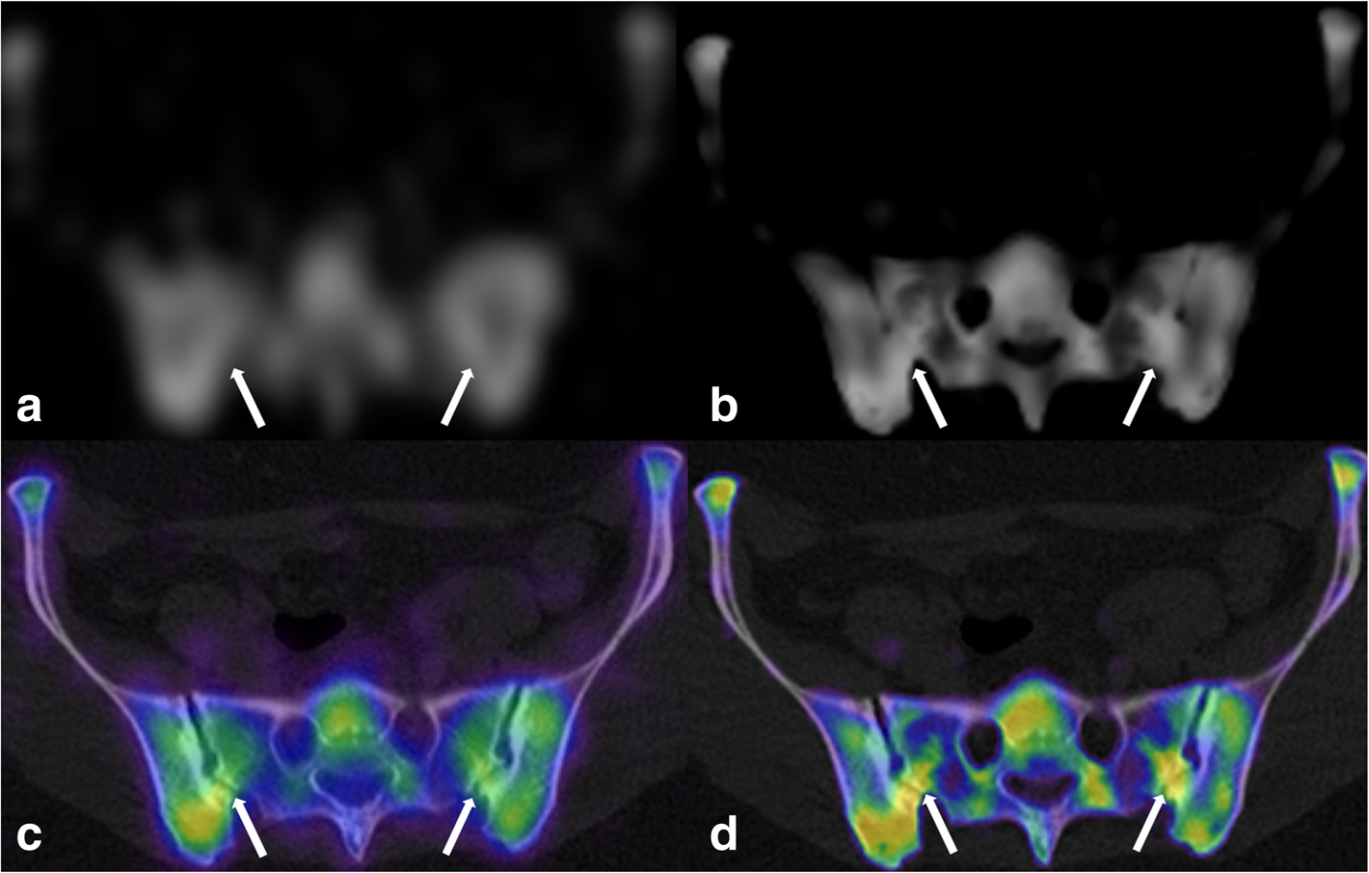
Osteoarthritis Left Posterior Sacroiliac Joints. Detailed Legend: 35 yr. old female with lower back pain and degenerative changes in posterior synovial sacroiliac joints (white arrows). Single transaxial slice (all same slice): **a** SPECT, **b** xSPECT, **c** SPECT-CT, and **d** xSPECT-CT. Note the difficulty in identifying any abnormality in the SPECT slice (**a**)

Figure 7 demonstrates the clear increase in bone definition in the pelvis with xSPECT/CT compared with SPECT. In the fused image the posterior sacroiliac joints are noted to be abnormal in configuration with secondary degenerative changes on the CT and demonstrate abnormal tracer uptake on xSPECT/CT but only equivocal changes on SPECT.

Low grade uptake was frequently identified in enthesial lesions which appear normal on SPECT. Unless severe, uptake in ossicles and sesamoid bones is rarely seen in SPECT, but was found in several (with negative SPECT/CT uptake) in this series.

In no case was malignancy or metastatic disease was identified only on the xSPECT/CT. In the 26 oncology cases the diagnosis was only changed in one case where the cause of pain was altered –it was non-malignant and the underlying status of metastatic disease was unchanged. However, the author has subsequently seen several cases where very early metastatic disease has been identified only on the xSPECT/CT. Figure 4 shows the potential to miss malignancy is higher on SPECT/CT but in that case the diagnosis was still made on the SPECT/CT, though principally from the CT rather than the SPECT images. The number of metastatic lesions identified was frequently greater on the xSPECT/CT than the SPECT/CT.

Interpretation problems were rare and included: variable prosthetic artefact, an increased number of clinically doubtful lesions, and difficulty confidently differentiating between reconstruction artefact and a significant finding –mostly related to small foci of calcification and very small lesions.

## Discussion

This study demonstrates that useful clinical information can be collected in the course of normal (solo) clinical practice. The methodology was simple with the use of commonly available online tools. The authors found the rigor of performing a structured new-old comparison was valuable mechanism of identifying and quantifying its strengths and weaknesses. However, the principal limitation in a solo practice is the difficulty in controlling reporter bias with no blinded second readings. Ideally the methodology would be greatly improved if a second or third reader was available. In an attempt to address this shortcoming, we included as much follow-up data, both clinical and imaging, as possible. In addition, the follow up blinded second reading supports the intra-reader reliability was high (particularly for SPECT/CT) and that read order was unlikely to be a significant factor in influencing the findings. The xSPECT second reads did show some discrepancy for non-significant findings, most likely related to accumulated reader experience with xSPECT/CT bone since the original reading. Reader bias as a result of learning from the initial SPECT/CT is also thought not likely to influence the results, as most often the significant differences between xSPECT/CT and SPECT/CT identified in this study, involve identifying additional lesions with xSPECT/CT, as demonstrated in some of the cases shown (Figs. 3, 4, 5, 6). These lesions remained undetected in the blinded second SPECT/CT read.

No statistical significance can be attributed to the quantity of diagnostic change seen in this observational study, but the substantial improvement in diagnostic confidence identified agrees with previous studies showing increased reader confidence with xSPECT/CT Bone scans compared with conventional SPECT/CT bone scans (Vija et al. 2014, 2015). The change in both final diagnosis and diagnostic confidence was only a slightly smaller magnitude than our earlier experience documenting the change from planar to SPECT/CT bone imaging (unpublished data from 2008 on 524 consecutive cases using the same methodology).

This study was undertaken in the context of a referral pattern consisting mostly musculoskeletal problems. The overall benefits identified for the musculoskeletal population may not be seen in an oncology setting. In the limited number (26) of cases with metastatic disease, more lesions were identified on xSPECT/CT Bone, but a change in disease status did not occur in these patients. This is in agreement with the recent Danish study which found that hybrid imaging (SPECT/CT, PET/CT and PET/MR) found more lesions than planar bone scans, but rarely changed the tumour staging (Löfgren et al. 2017). It should be noted however that our protocol for metastatic disease during this study was for targeted SPECT/CT (based on whole-body scans) rather than whole body SPECT/CT, and a recent study has found the latter changed the diagnosis in 5.7% compared with the former (Rager et al. 2017).

Looking at the 40 cases where the diagnosis was altered (after reviewing the xSPECT/CT) in more detail it is evident that the anatomic localization of tracer uptake in xSPECT/CT leads to a higher resolution that in turn often demonstrates more uptake in specific structures. This leads to much clearer definition of uptake around joints, endplates, entheses, ossicles, calcifications, etc. In the case of small structures this can make a substantial clinical difference.

The improvement in diagnostic confidence is not only associated with a greater number of lesions but in the confidence related to a negative scan for assessing potential metastatic disease, sacroiliitis, enthesitis, and bone stress lesions. In all joint abnormalities, the pattern of uptake is more defined and was found to more closely correlate with the MRI findings in the cases where follow-up was available. In the thoracic spine xSPECT uptake was frequently identified in the small joints of the spine and these are infrequently identified as abnormal on SPECT/CT imaging. These joints can be a significant cause of symptoms and in 6 cases lesions identified only on xSPECT/CT were treated with CT guided injections that led to relief of symptoms. xSPECT/CT appears to be an improved method of assessing difficult thoracic and chest pain.

It needs to be emphasised that the change in 40 report diagnoses does not indicate that the xSPECT/CT diagnosis is correct, but that additional information obtained from the scan prompted a change in interpretation by the reader. The follow-up data was obtained for verification was only available in 21(53%) of these cases (Table 1) and an independent diagnostic imaging standard was available for only 3 (8%). However, in only 2 or 3 of the 21 follow-up cases was the new diagnosis shown to be incorrect. Two of these were cases of sacroiliitis diagnosed by xSPECT/CT (but not SPECT/CT) that were negative on MRI. MRI has been shown to be insensitive to sacroiliitis (Goupille et al. 2009; Song et al. 2008) and it is likely only one of these was a false positive as the other was successfully treated as an inflammatory sacroiliitis. This study certainly suggests caution is needed in evaluating low level sacroiliac joint uptake on xSPECT/CT. While it may improve sensitivity for sacroiliitis this may be at the cost of specificity. The third identified possible diagnostic error was a patient with an abnormal thoracic costovertebral joint on xSPECT/CT that was not helped by a CT guided injection.

The greater resolution and the increased number of lesions seen with xSPECT/CT is a challenge to the reporting physician. It is therefore more important to correlate the increased number of findings with the patient’s specific clinical setting. In addition, the physician new to xSPECT/CT Bone needs to develop a new understanding of the normal variations seen with this reconstruction algorithm.

## Conclusion

This study suggests that xSPECT/CT Bone reconstruction offers identifiable imaging improvements over standard SPECT/CT reconstruction algorithms, and these or often clinically significant, particularly for musculoskeletal applications. It gives the reader more detail and increased diagnostic confidence with the potential to improve diagnostic accuracy. Further blinded and multi-reader studies are needed to determine whether this leads to a significant change in diagnostic performance. Further specific studies might quantify whether xSPECT has the potential to change the diagnostic role of technetium bone imaging in some clinical scenarios, such as the diagnosis of sacroiliitis.
